# Deregulation of PAX2 expression in renal cell tumours: mechanisms and potential use in differential diagnosis

**DOI:** 10.1111/jcmm.12090

**Published:** 2013-07-26

**Authors:** Patrícia Patrício, João Ramalho-Carvalho, Pedro Costa-Pinheiro, Mafalda Almeida, João Diogo Barros-Silva, Joana Vieira, Paula Cristina Dias, Francisco Lobo, Jorge Oliveira, Manuel R Teixeira, Rui Henrique, Carmen Jeronimo

**Affiliations:** a Cancer Epigenetics Group, Research Center of the Portuguese Oncology Institute - PortoPorto, Portugal; b Department of Genetics, Portuguese Oncology Institute – PortoPorto, Portugal; c Cancer Genetics Group, Research Center of the Portuguese Oncology Institute - PortoPorto, Portugal; d Department of Pathology, Portuguese Oncology Institute – PortoPorto, Portugal; e Department of Urology, Portuguese Oncology Institute – PortoPorto, Portugal; f Department of Pathology and Molecular Immunology Institute of Biomedical Sciences Abel Salazar, University of PortoPorto, Portugal

**Keywords:** Renal cell tumours, PAX2, differential diagnosis, chromosome 10 monosomy, promoter methylation

## Abstract

Expression of *PAX2* (Paired-box 2) is suppressed through promoter methylation at the later stages of embryonic development, but eventually reactivated during carcinogenesis. Pax-2 is commonly expressed in the most prevalent renal cell tumour (RCT) subtypes—clear cell RCC (ccRCC), papillary RCC (pRCC) and oncocytoma—but not in chromophobe RCC (chrRCC), which frequently displays chromosome 10 loss (to which *PAX2* is mapped). Herein, we assessed the epigenetic and/or genetic alterations affecting *PAX2* expression in RCTs and evaluated its potential as biomarker. We tested 120 RCTs (30 of each main subtype) and four normal kidney tissues. Pax-2 expression was assessed by immunohistochemistry and *PAX2* mRNA expression levels were determined by quantitative RT-PCR. *PAX2* promoter methylation status was assessed by methylation-specific PCR and bisulfite sequencing. Chromosome 10 and *PAX2* copy number alterations were determined by FISH. Pax-2 immunoexpression was significantly lower in chrRCC compared to other RCT subtypes. Using a 10% immunoexpression cut-off, Pax-2 immunoreactivity discriminated chrRCC from oncocytoma with 67% sensitivity and 90% specificity. *PAX2* mRNA expression was significantly lower in chrRCC, compared to ccRCC, pRCC and oncocytoma, and transcript levels correlated with immunoexpression. Whereas no promoter methylation was found in RCTs or normal kidney, 69% of chrRCC displayed chromosome 10 monosomy, correlating with Pax-2 immunoexpression. We concluded that Pax-2 expression might be used as an ancillary tool to discriminate chrRCC from oncocytomas with overlapping morphological features. The biological rationale lies on the causal relation between Pax-2 expression and chromosome 10 monosomy, but not *PAX2* promoter methylation, in chrRCC.

## Introduction

Renal cell tumours (RCTs) are the most common kidney neoplasms in adults, accounting for 2–3% of all non-cutaneous malignant neoplasms, ranking 14th in incidence for both genders and presenting a mortality rate of 1.6/100,000 worldwide [1]. Globally, in 2008, ∼167,947 new cases of kidney cancer were diagnosed and 72,030 patients died because of this malignancy [1]. Renal cell tumours are a heterogeneous group of neoplasms which arise from the epithelium of renal tubules [2] and represent more than 90% of all renal tumours occurring in adults in both genders [3]. The most prevalent benign RCT is oncocytoma, whereas among the malignant RCTs, clear cell renal cell carcinoma (ccRCC, 70–75% of cases), papillary renal cell carcinoma (pRCC, 10–15% of cases) and chromophobe renal cell carcinoma (chrRCC, 5–10% of cases) are the most common subtypes [3, 4]. Most RCTs display characteristic histological features, enabling their distinction in haematoxylin and eosin stained slides, but in a proportion of cases there are overlapping characteristics, which impair a clear diagnostic discrimination. For instance, the eosinophilic variant of chrRCC may be difficult to discriminate from oncocytoma, yet this distinction is critical because the former is a malignant tumour and the latter is a benign one, thus entailing different clinical approaches [5].

The *PAX2* (*Paired-box 2*) gene encodes a transcription factor from the evolutionarily conserved paired-box family, characterized by the presence of a paired-box DNA-binding domain [6]. This gene is located at 10q24.3-q25.1 and contains 12 exons [7], several of which are alternatively spliced, originating five well characterized alternative splice variants, sharing a common promoter region [8]. The *PAX2* gene product is required during development of the central nervous system and genitourinary tract [9], being expressed in the developing kidney, as well as in the optic stalk, midbrain–hindbrain junction, and spinal cord [10]. During kidney development, Pax-2 expression is primarily present during early stages of embryogenesis, where it is thought to suppress apoptosis and promote epithelial aggregation, then it is switched off in most part of the kidney structures in the later phases of differentiation [11]. Indeed, in adult kidney, *PAX2* mRNA expression is restricted to cells of the collecting ducts (where it seems to protect medullary cells against the stress induced by high levels of NaCl and urea) and, to a lesser extent, to cells of the distal tubules [12]. Although the precise mechanism of Pax-2 expression downregulation is not fully understood, epigenetic silencing mediated by *PAX2* promoter methylation has been reported in the normal adult kidney cells of rats [13]. Conversely, *PAX2* promoter methylation has been found in normal endometrium lacking Pax-2 expression and its loss has been associated with Pax-2 re-expression in endometrial carcinoma [14].

This temporal and spatial expression of *PAX* genes seems to be essential for Pax proteins function as nuclear transcription factors, critically involved in cell proliferation, differentiation and migration, and constitute, therefore, putative targets of disruption during tumourigenesis [15]. Although there is evidence for re-expression of the *PAX2* gene in RCTs, the mechanism of oncogenic activation has not been reported [16]. Indeed, significant Pax-2 protein expression has been found in three of the most prevalent RCT subtypes—clear cell RCC, papillary RCC and oncocytoma—but not in chromophobe RCC [17]. Interestingly, chromophobe RCC frequently display chromosome 10 loss [18, 19], which might justify that finding.

To clarify the mechanism(s) underlying *PAX2* deregulation in RCTs, we assessed *PAX2* mRNA and protein expression, as well genomic copy number changes and gene promoter methylation status in a large series of RCTs, representing the four major subtypes. Furthermore, the potential usefulness of Pax-2 immunoexpression for discriminating among RCTs subtypes was determined.

## Materials and methods

### Patients and sample collection

One hundred and twenty RCT samples, comprising 30 cases of each of the four major subtypes (ccRCC, pRCC, chrRCC, and Oncocytoma) were consecutively selected from a larger series of patients diagnosed and treated at the Portuguese Oncology Institute,Porto, Portugal, whom underwent partial or radical nephrectomy, after informed consent. In each case, a tumour fragment was immediately snap-frozen, stored at −80°C, and subsequently cut in a cryostat for nucleic acid (DNA and RNA) extraction. The bulk material was routinely fixed in buffered formalin and paraffin-embedded. The corresponding hematoxylin-eosin stained sections were examined by a pathologist to determine the tumour type [3], grade classification [20] and pathological stage according to the TNM staging system [21]. A representative paraffin block of each tumour was further selected for immunohistochemical and FISH analyses. Relevant clinical and pathological data were collected and are provided in Table 1.

**Table 1:** Clinical and pathological features of patient population

Clinicopathological features	RCT
Patients, *n*	120
Gender, *n* (%)	
Male	70 (58)
Female	50 (42)
Median age, years (range)	59 (29–83)
Pathological stage*, *n* (%)	
I	50 (56)
II	22 (24)
III	16 (18)
IV	2 (2)
Furhman grade*, *n* (%)	
1	2 (2)
2	29 (32)
3	39 (44)
4	20 (22)

* Includes RCC cases only.

These studies were approved by the ethics committee of Portuguese Oncology Institute – Porto (Comissão de Ética do IPO Porto).

### Nucleic acids extraction and cDNA synthesis

Genomic DNA was extracted from tumour and normal tissues by the phenol–chlorophorm method at pH 8, as previously described [22]. Samples were first submitted to overnight digestion, in a bath at 55°C, using buffer solution SE (75 mM NaCl; 25 mM EDTA), SDS 10% and proteinase K, 20 mg/ml (Sigma-Aldrich®, Schnelldorf, Germany). After digestion, extraction was performed with phenol/chloroform (Sigma-Aldrich®, Schnelldorf, Germany, Merck, Darmstadt, Germany) followed by precipitation with 100% ethanol.

Total RNA from RCT samples was extracted by the PureLink™ RNA Mini Kit (Invitrogen, Carlsbad, CA, USA) and reverse transcription was accomplished by using the RevertAid™ H Minus First Strand cDNA synthesis Kit (Fermentas, Ontario, Canada), following the manufacturer's instructions.

DNA and RNA quality and concentration were analysed in a NanoDrop ND-1000 (NanoDrop Technologies, DE, USA) spectrophotometer.

### *PAX2* mRNA quantification

Quantification of *PAX2* mRNA was performed by real-time RT-PCR using Taqman probes on an Applied Biosystems 7500 Real-time PCR (Applied Biosystems, Foster City, CA, USA). Primers and probes for *PAX2* (Hs01057413_m1) and *HPRT1* (Hs 99999909_m1) (endogenous control) were purchased as pre-designed optimized real-time PCR assay reagents from Applied Biosystems. In each reaction, 9 μl of cDNA, 1 μl of Taqman® Gene Expression Assay and 11 μl of Taqman® Gene Expression Assay Master Mix (Applied Biosystems) were used. All samples were run in triplicate. Two negative controls, consisting on the replacement of cDNA with DEPC-treated water (MP Biomedicals, Solon, OH, USA), were included in each plate. PCR parameters were as follows: 50°C for 2 min., 95°C for 10 min. and 45 amplification cycles at 95°C for 15 sec. and 60°C for 1 min. For quantitation, a standard curve was built with a series of cDNA dilutions prepared from commercial Stratagene® QPCR Human Reference total RNA (Agilent Technologies, Stratagene, La Jolla, CA, USA) or from a *PAX2* expressing RCT (oncocytoma), for *HPRT1* and *PAX2* analysis, respectively. *PAX2* expression levels were calculated by dividing the values of *PAX2* mean quantity by those of *hypoxanthine phosphoribosyltransferase 1 (HPRT1),* as endogenous control. This ratio was then multiplied by 1000 for easier tabulation.

### Immunohistochemistry

Immunohistochemistry was performed according to the avidin-biotin method using the VECTASTAIN® Universal Elite ABC Kit (©Vector Laboratories, Burlingame, CA, USA). Four micron-thick sections from the representative paraffin block of each RCT and of five normal endometrium samples (collected from women submitted to hysterectomy for uterine leiomyomas, and intended as controls) were deparaffinized with xylene and rehydrated with consecutive ethanol solutions (100%, 90% and 70%). Subsequently slides were steamed in a 1× sodium citrate buffer solution (©Vector Laboratories) for 20 min. in a 700 W microwave oven for antigen retrieval. Endogenous peroxidase was blocked by incubating the slides in a 0.6% H_2_O_2_ solution for 20 min. After washing the slides in distilled water and PBS/tween 20 solution, they were incubated with Normal Horse Serum 1:100 in PBS-BSA for 30 min., in a humid chamber, at room temperature. Then, slides were incubated with primary antibody [rabbit monoclonal antibody, Pax-2 (clone ab79389; Abcam, Cambridge, UK)] at a dilution of 1:3000 for 18 hrs, at 4°C, in a humid chamber. The slides were next incubated with a biotynilated secondary antibody 1:100 in PBS/BSA for 30 min. in a humid chamber, at room temperature and localization was performed by incubating the sections with the ABC complex 1:100 in PBS/BSA for 45 min., in a humid chamber, at room temperature. The slides were then immersed in a 3,3′-diaminobenzidine solution for 7 min. Slides were counterstained with hematoxylin for 10 sec., washed in tap water, dehydrated, and mounted with Entellan® (Merck, Germany). An unrelated ccRCC section showing intense immunoreactivity for Pax-2 protein was used as positive control. The negative control consisted on the omission of the primary antibody.

The results for Pax-2 expression in each tumour were expressed in a semi-quantitative approach according to the estimated percentage of positive tumour cells. Immunostaining of more than 10% of the tumour cell nuclei was required for scoring a case as positive. The positive cases were further divided into two categories according to the proportion of positive cells: >10–50% of the nuclei stained and >50% of the nuclei stained [17, 19, 23].

### Bisulfite modification and methylation-specific PCR

DNA samples from RCTs, normal renal tissue (NK, four cases obtained from patients without renal tumour, used as negative control for methylation status) and normal endometrium (two cases, serving as putative positive control for *PAX2* promoter methylation [14]), were subjected to sodium bisulfite modification, using the EZ DNA Methylation-Gold™ Kit (Zymo Research, Orange, CA, USA) according to manufacturer's instructions.

Methylation analysis of *PAX2* promoter was performed by methylation-specific PCR (MSP). Bisulfite-modified genomic DNA was tested using two sets of primers, each recognizing either the methylated or the unmethylated sequences located at the promoter region of *PAX2* gene. Two adjacent regions were screened to assess the promoter methylation status [13, 14]. Primer sequences are summarized in Table 2, along with amplicon lengths and positions, and also optimized annealing temperatures.

**Table 2:** Sequences of the primers used in the methylation-specific PCR assays

Primer	Primer sequence (5′–3′)	AS (bp)	AL	AnT (ºC)	Reference
PAX2_M (F1)	GGGTTTTTTTCGTCGAAGTTC	170	−676 to −846	60	[14]
PAX2_M (R1)	ACTAAAACCTCGACTCCCGAT				
PAX2_U (F1)	GGTTTTTTTTGTTGAAGTTTGG	172	−673 to −845	62	[14]
PAX2_U (R1)	AAAACTAAAACCTCAACTCCCAAT				
PAX2_M (F2)	AGTTGTTAGCGTCGTTCGGTTT	139	−489 to −628	62	[13]
PAX2_M (R2)	ACAATCCCGAAAATATCCGAAATAA				
PAX2_U (F2)	AGAGTTGTTAGTGTTGTTTGGT	141	−489 to −630	59	–
PAX2_U (R2)	ACAATCCCAAAAATATCCAAAAT				

AS: amplicons size; AL: Amplicons location in relation to the transcriptional start site; Ant: Optimized annealing temperatures; M: Methylated; U: Unmethylated; F: Forward; R: Reverse; bp: base pairs.

PCR reactions were performed in an Applied Biosystems Veriti® Thermal Cycler (Applied Biosystems). Each PCR tube contained 14.36 μl of sterile distilled water (B. Braun, Melsungen, Germany), 2 μl of 10× DyNAzyme™ Hot Start Reaction Buffer (Finnzymes, Espoo, Finland), 0.2 mM of dNTPs (Fermentas, Ontario, Canada), 250 nM of primer forward and 250 nM of reverse primer, 0.24 μl of DyNAzyme™ II Hot Start DNA polymerase 2 U/μl (Finnzymes, Finland), and 2 μl of sample DNA, to a final volume of 20 μl. PCR amplifications were performed as follows: 10 min. at 94°C, followed by 38 cycles of 30 sec. at 94°C, 30 sec. at annealing temperature (see Table 2) and 30 sec. at 72°C. A 10 min. elongation step at 72°C completed the PCR amplification and PCR products were then submitted to electrophoresis on a 2% agarose gel.

Primers' specificity was assessed using CpGenome™ Universal Methylated DNA (Millipore, Darmstadt, Germany) and CpGenome™ Universal Unmethylated DNA (Millipore) as positive and negative controls, respectively.

### Bisulfite sequencing

The promoter region of the *PAX2* gene was subjected to bisulfite sequencing in the aforementioned normal kidney and endometrial tissue samples. Primer sequences, amplicons, and annealing temperatures are listed in Table S1. PCR reactions included a 94°C denaturation 10 min. step followed by 40 cycles at 94°C for 30 sec., annealing temperature for 30 sec., and 72°C for 30 sec. PCR products were loaded in a 2% agarose gel, stained with ethidium bromide and visualized under an ultraviolet transilluminator. Excess primer and nucleotides were removed by Illustra GFX PCR DNA and Gel Band Purification kit (GE Healthcare, USB Corporation, Cleveland, OH, USA) following manufacturer's protocol. The purified products were sequenced using the dGTP BigDye Terminator Cycle Sequencing Ready Reaction kit (Applied Biosystems) in an ABI PRISMTM 310 Genetic Analyzer (Applied Biosystems). The approximate amount of methylcytosine of each CpG site was calculated by comparing the peak height of the cytosine signal with the sum of the cytosine and thymine peak height signals [24]. CpG sites with ratios 0–0.20, 0.21–0.80, and 0.81–1.0 were considered unmethylated, partially methylated, and fully methylated, respectively.

### Fluorescence *in situ* hybridization analysis

Bacterial Artificial Chromosome (BAC) clones targeting the *PAX2* gene (RP11_1061B5) were selected using the UCSC Human Genome Browser platform and obtained from the BACPAC Resources Center (Oakland, USA). The *Escherichia coli* bacteria were first grown in LB agarose medium supplemented with cloramphenicol 12.5 mg/ml, at 37°C overnight. Then, an individual colony was inoculated in 10 ml of liquid LB medium and incubated at 37°C for 16 hrs with continuous agitation. The culture was centrifuged and pellet used for plasmid DNA extraction using the NucleoSpin® Plasmid kit (Macherey-Nagel, Düren, Germany), according to manufacturer's instructions. DNA concentration was determined in a NanoDrop ND-1000 spectrophotometer.

After adjusting the plasmid DNA concentration to 10 ng/μl, amplification was carried out using the Illustra GenomiPhi V2 DNA Amplification Kit (GE Healthcare), according to manufacturer's instructions. Probes were labelled using a nick translation DNA labelling system (Enzo Life Sciences, Exeter, UK). DNA was eluted in 10 μl of Vysis LSI/WCP Hybridization Buffer (Abbott Molecular, Des Plaines, IL, USA).

FISH analysis for *PAX2* was performed in 4 μm thick tissue sections obtained from representative paraffin blocks of each sample and placed in SuperFrost Plus Adhesion slides (Menzel-Glaser, Braunschweig, Germany). Sample processing, hybridization, and analysis were performed as previously described [25]. The *PAX2* probe, labelled with SpectrumGreen, was combined with the Vysis centromeric probe for chromosome 10 (CEP10), labelled with SpectrumOrange (Abbott Molecular) and applied to each sample. For scoring purposes, only intact, non-overlapping nuclei were considered. An abnormal population was considered representative when at least 10% of neoplastic cell nuclei presented a copy number change.

### Statistical analysis

The chi-square test or Fisher's exact test were used to uncover differences in the frequency of immunoreactivity for Pax-2 protein according to immunohistochemical scoring, among the four RCT types. To assess the value of Pax-2 protein immunohistochemical expression for discrimination between chromophobe RCC and oncocytomas, sensitivity, specificity, positive and negative predictive values (PPV and NPV) were determined using the 10% or the 50% cut-off values. Differences among groups were assessed by the Kruskal–Wallis non-parametric test, followed by pairwise comparisons using the Mann–Whitney U test, when appropriate. Association between *PAX2* mRNA expression levels and protein expression (immunohistochemistry score) was carried out using the Mann–Whitney U test. Relation between FISH and immunoexpression results was assessed by chi-square or Fisher's exact test, and the directional measure Somers'd was also computed. Moreover, the association between FISH findings and *PAX2* mRNA levels for chrRCC was estimated using the Kruskal–Wallis test. Significance value was set at *P* < 0.05 and Bonferroni's correction was used when appropriate. All tests were two-sided. Statistical analysis was performed using SPSS for Windows, version 15.0 (SPSS, Chicago, IL, USA).

## Results

### *PAX2* expression by quantitative RT-PCR

**Fig. 1:**
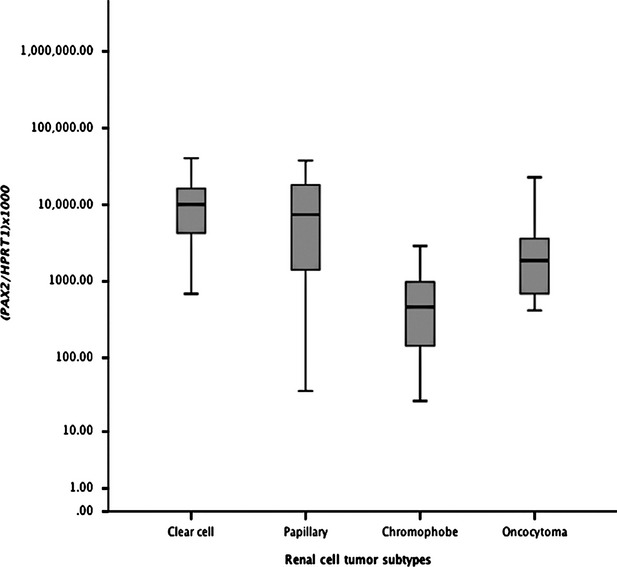
Distribution of *PAX2* relative mRNA expression levels (Log 10 transformed) of in renal cell tumours according to histological subtype (*n* = 120).

Significant differences were found for *PAX2* mRNA gene expression among the four RCT subtypes (Kruskal–Wallis test, *P* < 0.001, Fig. 1). Although *PAX2* mRNA expression levels did not differ between ccRCC and pRCC, both were significantly higher when compared to chrRCC and oncocytoma (*P* < 0.001, except for papillary *versus* oncocytoma: *P* = 0.011). Moreover, mRNA expression levels in oncocytomas also differed significantly from those of chrRCC (*P* < 0.001). *PAX2* mRNA expression levels differed significantly among Fuhrman grade categories (*P* < 0.001). Grade 4 tumours displayed the lowest relative median levels and these statistically differed from those of grade 2 and grade 3 tumours (*P* < 0.01, for both). However, no correlation was found between *PAX2* mRNA expression levels and patients' age (*r* = −0.112, *P* = 0.295) or pathological tumour stage (*P* = 0.542).

### Pax2 protein expression by immunohistochemistry

**Fig. 2:**
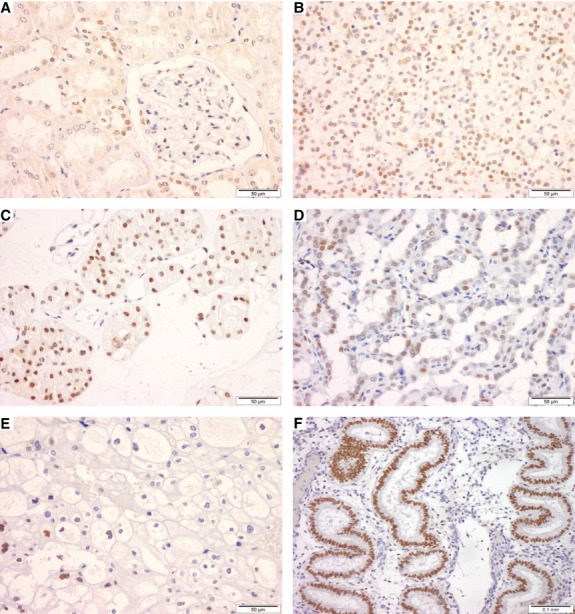
Pax-2 protein expression by immunohistochemical analysis in normal kidney (**A**), and endometrial glands (**F**) and renal cell tumours (**C**–**E**). Both normal distal tubules and the collecting duct cells with intense homogeneous nuclear immunoreactivity (**A**). Higher intensity staining was depicted in ccRCC (**B**) and oncocytomas (**C**), as well as in pRCC (**D**), whereas most of chrRCC were negative (**E**).

As expected, immunoreactivity for Pax-2 was only found in cell nuclei. In normal renal tissue, Pax-2 immunoexpression was restricted to distal tubules and collecting duct cells, which showed intense and homogeneous staining (Fig. 2A). Among RCTs, intense staining was shown by ccRCC (Fig. 2B), oncocytomas (Fig. 2C), and pRCC (Fig. 2D), whereas most chrRCC were negative (Fig. 2E). The results of immunostaining scoring for Pax-2 in all RCT subtypes are summarized in Table 3.

**Table 3:** Immunohistochemical expression of Pax-2 in histological sections of renal cell tumours (RCTs)

RCT subtype	Negative, *n* (%)	Positive, *n* (%)	
	
	≤10%	>10–50%	>50%
Clear cell (*n* = 30)	1 (3.3)	4 (13.4)	25 (83.3)
Papillary (*n* = 30)	13 (43.3)	4 (13.4)	13 (43.3)
Chromophobe (*n* = 30)	20 (66.7)	6 (20)	4 (13.3)
Oncocytoma (*n* = 30)	3 (10)	5 (16.7)	22 (73.3)

In normal endometrium samples, intense immunostaining for Pax-2 was apparent in all nuclei of endometrial glands (Fig. 2F) in all cases tested (*n* = 5).

**Fig. 3:**
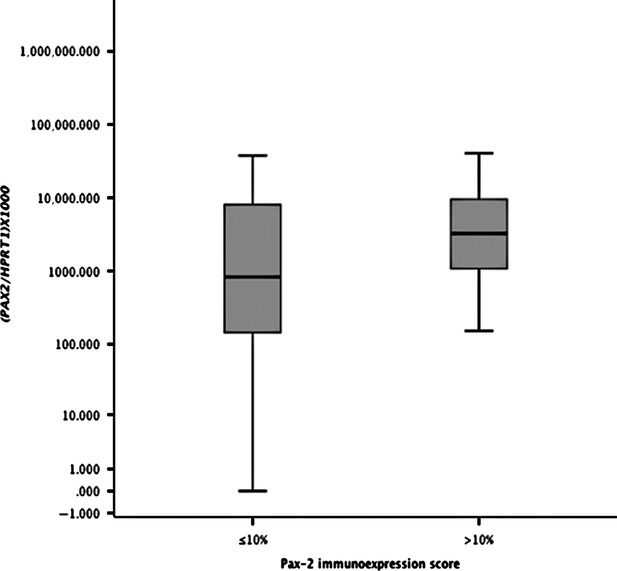
Distribution of *PAX2* mRNA expression levels (Log 10 transformed) according to Pax-2 immunoexpression scoring using the 10% cut-off value (*n* = 120).

Statistically significant differences were observed for quantitative Pax-2 expression among the four subtypes of RCTs (chi-square test, *P* < 0.001). Pairwise comparisons disclosed significant differences in all cases (*P* < 0.01) except for pRCC *versus* chrRCC (*P* = 0.119) and for oncocytoma *versus* ccRCC (*P* = 0.612). Concerning the clinicopathological data, we found significant differences between the immunoexpression frequencies according to Furhman grade (*P* = 0.04) and pathological stage (*P* = 0.03). These results are shown in Table 4. Both grade and stage were grouped in two categories for statistical purposes (Fig. 3).

**Table 4:** Correlation between PAX2 immunoexpression and Furhman nuclear grade and pathological tumour stage, *n* (%)

	Immunoexpression scoring		*P*
	
	≤10%	>10%	
Furhman grade			
1–2	7 (23%)	24 (77%)	0.04
3–4	27 (46%)	32 (54%)	
Stages			
I–II	23 (32%)	49 (68%)	0.03
III–IV	11 (61%)	7 (39%)	

**Fig. 4:**
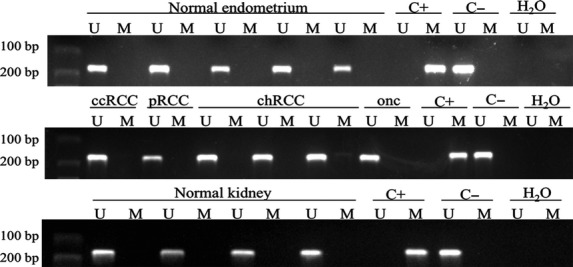
Representative methylation-specific PCR results from the analysis of *PAX2* promoter region (−676 to −846; primer set 1) in renal cell tumours, normal kidney and endometrium tissues using primers published by Wu *et al*. A visible PCR product in lanes U indicates the presence of unmethylated alleles whereas a PCR product in lanes M indicates the presence of methylated alleles. C+: fully methylated DNA, positive control for methylated samples; C−: fully unmethylated DNA, positive control for unmethylated samples; H_2_O: negative control. U: lane for unmethylated MSP; M: lane for methylated MSP.

Pax-2 negative cases, categorized according to the 10% cut-off value, showed significantly lower median mRNA levels compared to Pax-2 positive cases (*P* = 0.011), as illustrated in Figure 4.

Because the morphological differential diagnosis between chrRCC and oncocytoma might be troublesome, we tested whether Pax-2 immunostaining might be used as an ancillary tool for histopathological assessment. Indeed, the best Pax-2 immunostaining cut-off value for discriminating between chrRCC and oncocytoma was 10%, with a sensitivity and specificity of 67% and 90%, respectively. For comparison purposes, we also calculated the performance of Pax-2 immunoexpression for discriminating chrRCC from oncocytoma in previous publications, and the results are summarized in Table 5.

**Table 5:** Performance of Pax-2 immunoscoring for the discrimination between chromophobe RCC and oncocytoma

	Sensitivity (%)	Specificity (%)	PPV (%)	NPV (%)
Present study				
10% cut-off	67	90	87	73
50% cut-off	87	73	76	85
Gupta *et al*. ([17])				
10% cut-off	94	100	100	85
50% cut-off	94	65	89	79
Mazal *et al*. ([23])				
10% cut-off	91	10	50	54
50% cut-off	100	3.5	51	100
Memeo *et al*. ([19])				
10% cut-off	91	87	77	95

PPV: Positive predictive value; NPV: negative predictive value.

### *PAX2* promoter methylation

**Fig. 5:**
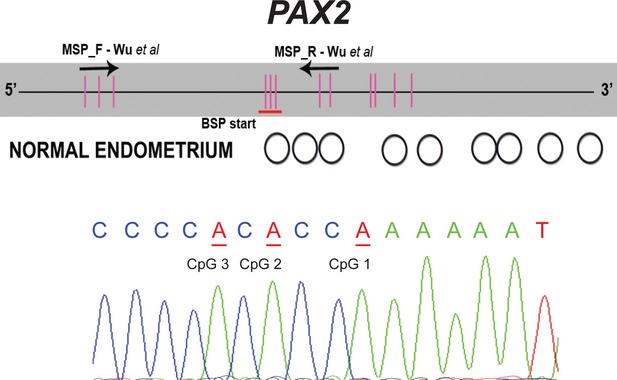
Characterization of the methylation status of individual CpG dinucleotides by bisulfite sequencing of the *PAX2*. The upper part of each panel provides a schematic representation of the CpG island in the transcription start (+1) region. Vertical bars indicate the location of individual CpG sites and the two arrows indicate a location of methylation-specific PCR primers. For the middle part of the panel, unfilled circles represent unmethylated CpGs, black-filled circles represent methylated CpGs and grey-filled circles represent partially methylated sites. The lower panel is a section of the bisulfite reverse sequence electropherogram, were cytosines in CpG sites are in underlined red as adenines due to bisulfite conversion.

Using the previously published set of primers by Wu *et al*. no methylation was found for the *PAX2* promoter in any of the tumour or normal renal tissue samples. As these primers showed *PAX2* promoter as methylated in normal endometrium [14], we tested the same primer's set in two normal endometrium tissue samples and both were unmethylated at the *PAX2* promoter (Fig. 5).

**Fig. 6:**
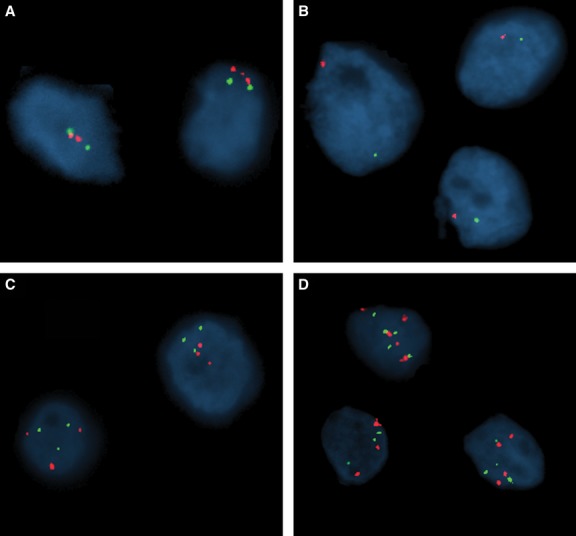
*PAX2*/CEP 10 copy number changes in chrRCC (chromosome 10 probe in red; *PAX2* probe in green). (A) Normal. (B) Monosomy. (C) Trisomy. (D) Polysomy.

Because no methylation was found, either in RCTs or in normal tissues using the two primer sets and *PAX2* methylation might occur at regions that were not assessed by MSP, we performed bisulfite sequencing of the entire *PAX2* promoter region CpG island. Strikingly, the full characterization of the methylation status of *PAX2* promoter allowed us to confirm absence of methylation in most of CpG dinucleotides within the CpG island (Fig. 6).

### *PAX2*/Chromosome 10 copy number alterations

All 30 chrRCC cases were tested for *PAX2*/chromosome 10 copy number alterations using FISH probes targeting the *PAX2* gene and chromosome 10 centromeric region, respectively. Chromosome 10 loss was observed in 18 (69%) tumours, whereas gain was detected in two (8%) cases, and no alterations were found in six (23%) out of the 26 chrRCC analysable (four chrRCC samples were not included due to technical reasons). Interestingly, no isolated *PAX2* locus deletion was found in any of the examined samples. Figure 6 shows representative FISH images of chrRCC tested.

Significant differences in Pax-2 immunoexpression were observed among the three categories of *PAX2*/CEP 10 copy number changes, when the 10% (*P* < 0.001) or the 50% (*P* = 0.018) cut-off values were used (Table 6). Moreover, Somers'd directional measure revealed a strong positive association between the immunoexpression frequency and *PAX2*/CEP 10 copy number changes, both using the 10% (*d* = 0.8, *P* < 0.001) or 50% (*d* = 0.498, *P* = 0.041) immunoexpression cut-off values. No statistically significant association was found between *PAX2*/CEP 10 copy number and *PAX2* mRNA expression (*P* = 0.919).

**Table 6:** FISH results for *PAX2*/CEP10 copy number and distribution of the immunoexpression frequencies and mRNA expression levels, in chrRCC cases

FISH analysis	Immunoexpression, *n* (%)				mRNA expression Median (interquartile range)

	10% cut-off		50% cut-off		
	
	−	+	−	+	
Monosomy	17 (94)	1 (6)	18 (100)	0 (0)	213.2 (104.9–1000.4)
No ch10 loss	1 (17)	5 (83)	4 (67)	2 (33)	524.7 (114.1–1508.3)
Polysomy	0 (0)	2 (100)	1 (50)	1 (50)	384.7 and 527.7*

* Descriptive values.

## Discussion

*PAX2* is an important transcription factor intensely expressed during kidney development [10], but which is downregulated in the latter phases of differentiation in most of the kidney structures [11]. In the mature kidney, Pax-2 expression is restricted to the collecting ducts and distal tubules, where it promotes osmotic tolerance and protects cells from apoptosis due to high sodium chloride and urea exposure [12]. The mechanisms leading to *PAX2* silencing at the end of kidney development, however, are still controversial and hypotheses include *PAX2* promoter methylation [13] and WT1 mediated repression [26]. Moreover, *PAX2* has been reported to function as an oncogene, conferring proliferative and apoptosis inhibitory characteristics to cells in several tumour models [16], including RCTs [27]. Finally, diffuse protein expression of Pax-2 has been reported to be frequent in three of the most prevalent RCT subtypes - ccRCC, pRCC and oncocytoma - but not in chrRCC, suggesting its use for differential diagnosis in problematic cases [17, 19, 23, 28].

In this study, immunohistochemical analysis of Pax-2 expression disclosed significant differences among the four major subtypes of RCT. Whereas ccRCC and oncocytoma showed the highest frequency of positive cases (immunoexpression >10% of tumour cells), chrRCC showed the opposite trend, in line previous reports [17, 19, 23], although some differences were apparent mainly in the frequency of positive cases among chrRCC. As the same cut-off value was used, it is likely that methodological differences underlie these discrepant results. Indeed, we used a monoclonal antibody (which tends to be more specific), in a 1/3000 dilution, whereas all the aforementioned studies used polyclonal antibodies in 1/50 and 1/100 dilutions [17, 19, 23]. Thus, the higher frequency of Pax-2 immunoreactivity in chrRCC found in our study is not due to lack of antibody specificity or to excessive concentration.

Some degree of morphological overlap exists between some RCC subtypes, which prompted the use of panels of immunohistochemical markers to assist the diagnosis in difficult cases [29]. Although several panels have been proposed, none has reached widespread acceptance. Thus, we also aimed to confirm the potential of Pax-2 as a biomarker for discrimination between RCT subtypes, specifically between chrRCC (a malignant tumour) and oncocytoma (a benign tumour), which might be difficult to discriminate morphologically, despite being distinct entities with different prognosis and clinical behaviour. For that purpose, we determined the validity estimates using the 10% or the 50% cutoffs and compared with previously published data [17, 19, 23]. Our results followed the same trend of those studies, confirming that the 10% cut-off value offers the highest specificity, whereas, as expected, the 50% cut-off value displayed the highest sensitivity. The more discrepant values are those of Mazal *et al*. as they only reported Pax-2 positivity in 14% of oncocytomas, against 87–100% of positivity in our study and those of Memeo *et al*. and Gupta *et al*. [17, 19, 23].

In line with the observations of Pax-2 protein expression, *PAX2* mRNA expression also unveiled significant differences among the four RCT subtypes. However, the highest levels were observed in pRCC and ccRCC, whereas oncocytomas showed intermediate levels, and chrRCCs displayed the lowest levels of *PAX2* mRNA. Importantly, associations between *PAX2* mRNA levels and protein immunoexpression were also apparent, using both immunohistochemical cut-off values above referred, implying that *PAX2* transcription and translation are correlated. The differences in Pax-2 expression between pRCC and ccRCC on one hand, and chrRCC and oncocytoma, on the other, may be related with the site of origin of each tumour type and may, thus, reflect distinct carcinogenic pathways. Indeed, whereas the former derive from proximal tubule cells, the latter originate from cortical collecting duct cells [30]. This is a somewhat unexpected finding because in normal renal tissue, Pax-2 expression is restricted to the distal portion of the nephron. Taken together, these findings suggest that Pax-2 is overexpressed in pRCC and ccRCC, whereas in chrRCC it is underexpressed, when compared to their normal cell counterparts. Hypothetically, oncocytomas retain the normal Pax-2 expression patterns. This model is in accordance with the putative proto-oncogenic function of the *PAX2* gene [16], as pRCC and ccRCC are malignant neoplasms and oncocytoma is a benign tumour. Therefore, the loss of Pax-2 expression in chrRCC might be interpreted as a bystander alteration and it is tempting to speculate whether this might contribute to the less aggressive clinical behaviour of chrRCC compared to pRCC and ccRCC [31].

Correlation analyses revealed similar trends in PAX2 expression at the protein or transcript levels and tumour grade, disclosing significant inverse correlations. Although these findings were somewhat unpredicted owing to the role ascribed to *PAX2* as a transcription factor, our results are in agreement with those of Mazal *et al*., who also reported a decrease or lack of Pax-2 immunoexpression with increasing Fuhrman grade [23], and of Luu *et al*., although a different grading system was used and only ccRCC were analysed [32]. Concerning pathological tumour stage, however, only PAX2 immunoexpression showed a significant inverse correlation, whereas the same trend was not apparent at the mRNA level. The reason for this discrepancy is not immediately apparent and may derive from the higher heterogeneity of the staging system compared to the more homogeneous nuclear grading system.

Another main objective of the present study was to ascertain the mechanism underlying the differential expression of Pax-2 among RCT subtypes. Two possibilities for *PAX2* downregulation have been suggested: *PAX2* promoter hypermethylation and/or gene deletion. The former possibility was founded upon existence of a CpG island in the promoter region and the putative role of promoter methylation in *PAX2* gene silencing at the final stages of kidney development [13]. The latter hypothesis stemmed from the observation that loss of chromosome 10 (to which *PAX2* is mapped) is a frequent event in chrRCC [17, 19, 23].

To test the *PAX2* promoter methylation hypothesis, methylation-specific PCR was performed not only in chrRCC but also in the remainder RCT subtypes and normal kidney tissue samples, using two different sets of primers that anneal to two adjacent regions in the promoter [13, 14]. These regions have been previously reported to be involved in *PAX2* gene transcription regulation. Remarkably, no methylation was detected in those regions either in any of the analysed RCT samples, including chrRCC nor in normal kidney tissues. Furthermore, no methylation was found in normal endometrium (five random samples), which was previously reported to harbour *PAX2* promoter methylation using one of the primer sets [13, 14]. In addition, we tested for methylation using the other set of primers and the result was again negative. Importantly, we demonstrated Pax-2 protein expression in endometrial glands [which were negative according to previous studies [13, 14], which is consistent with absence of methylation by MSP analysis. Moreover, our results are in accordance with those of Luu *et al*., which also did not find *PAX2* promoter methylation in ccRCC or in normal kidney tissues [32]. As bisulfite sequencing allows for a more detailed characterization of the methylation status for individual CpGs, we used this approach to further confirm the absence of methylated residues in the promoter region of *PAX2*. Bisulfite sequencing in normal kidney and endometrium samples validated MSP results, showing that no methylation was present in *PAX2* promoter region. Taken together our results indicate that methylation does not explain *PAX2* different expression levels both when comparing groups of tumours and stages of kidney development.

To explore the chromosome 10 loss hypothesis, FISH analysis using specific probes for chromosome 10 and for the *PAX2* gene was performed in all cases of chrRCC. As expected, the vast majority of chrRCC cases exhibited chromosome 10 monosomy, which is in accordance with a previous report [18], and no case of isolated *PAX2* locus deletion was observed. This might explain both the lower *PAX2* mRNA levels and protein expression globally observed in this tumour subtype. Remarkably, a significant association with the immunoexpression findings was observed, indicating that chromosome 10 loss is the most likely mechanism underlying *PAX2* loss of expression. This is further supported by the higher frequency of Pax-2 immunoexpression in cases with no copy number change or with polysomy, a trend confirmed by the Somers'd test. Interestingly, the highest correlation value was found for the 10% immunoexpression cut-off, suggesting that this cut-off might be more biologically meaningful. Our results indicate that *PAX2* promoter methylation is not implicated in gene silencing in chrRCC or in normal kidney. Instead, the frequent chromosome 10 loss observed in chrRCC samples, and the significant correlation with Pax-2 immunoexpression categories, indicate that this genetic alteration is the major cause for PAX2 underexpression in this tumour subtype. Importantly, this feature might be used for discriminating chrRCC from oncocytoma in challenging cases. Nonetheless, additional studies are required to further determine the genetic and/or epigenetic regulation mechanisms underlying *PAX2* differential expression in other RCTs.
